# Neo-antigen specific T cell responses indicate the presence of metastases before imaging

**DOI:** 10.1038/s41598-019-51317-3

**Published:** 2019-10-10

**Authors:** V. S. Fear, C. A. Forbes, J. Chee, S. Ma, S. Neeve, L. Celliers, S. A. Fisher, I. Dick, J. Creaney, B. W. S. Robinson

**Affiliations:** 1 National Centre for Asbestos Related Diseases (NCARD), Lv5 QQ Block (M503), QEII Medical Centre, The University of Western Australia, Perth, Western Australia 6009 Australia; 2 School of Biomedical Sciences, The University of Western Australia, Perth, Western Australia 6009 Australia; 3 Australian Cancer Research Foundation, Cancer Imaging Facility, Perth, Western Australia 6009 Australia; 4 Centre for Respiratory Health, The University of Western Australia, Perth, Western Australia 6009 Australia; 5 School of Medicine, The University of Western Australia, Perth, Western Australia 6009 Australia

**Keywords:** Immunological techniques, Non-small-cell lung cancer, Tumour immunology

## Abstract

Non-small cell lung cancer (NSCLC) causes 19% of all Australian cancer deaths, with a 5-year survival post-resection of around 60%. Post-operative recurrence is due to metastases that were undetectable pre-operatively, or growth of microscopic locoregional residual disease. However, post-operative imaging modalities typically only detect more advanced tumours; where PET-CT has a detection limit of 6–7 mm. Detection of small deposits of lung metastatic disease is of importance in order to facilitate early and potentially more effective treatment. In this study, in a murine model of lung metastatic disease, we explore whether neo-antigen specific T cells are a sensitive marker for the detection of lung cancer after primary tumour resection. We determine lung metastatic disease by histology, and then compare detection by PET-CT and neo-antigen specific T cell frequency. Detection of lung metastatic disease within the histology positive group by PET-CT and neo-antigen specific T cell frequency were 22.9% and 92.2%, respectively. Notably, neo-antigen specific T cells in the lung draining lymph node were indicative of metastatic disease (82.8 ± 12.9 spots/10^5^ cells; mean ± SE), compared to healthy lung control (28.5 ± 8.6 spots/10^5^ cells; mean ± SE). Potentially, monitoring tumour neo-antigen specific T cell profiles is a highly sensitive method for determining disease recurrence.

## Introduction

Globally, more than 15 million cancer patients undergo resection of their tumour every year^1^. However, disease recurrence after surgery for many cancers is frequent, either due to the growth of metastases that were not detectable before the operation or due to progressive growth of microscopic residual disease^2–4^.

Immunotherapy is a breakthrough modality for the treatment of a number of cancers, and works best when tumour deposits are relatively small^5^. For example, in melanoma patients the efficacy of tremilimumab in treatment of stage III disease is 60%, compared to 17.7% in stage IV disease^6^. Similarly, in animal models, immunotherapy is most effective when tumour deposits are small^5,7^. Accordingly, earlier detection of metastatic disease may improve patient outcomes to immunotherapy.

In this study we focus on NSCLC, one of the most common lethal cancers. NSCLC has an incidence of around 43 cases per 100,000, and accounts for 19% of all cancer deaths^8^. 5-year survival after resection is approximately 60%^9^, indicating that current curative-intent treatment has limited efficacy and that residual microscopic tumour deposits remain undetected. Imaging modalities are typically used to detect the presence of metastatic disease, although it is known to miss small deposits. PET-CT is one of the main such imaging modalities and is routinely used in pre- and post-operative staging. Depending upon anatomical location, PET scanning has a tumour detection limit of 6–7 mm, with detection limits for CT at 3–4 mm for malignant masses. Given the proportion of patients who go on to develop metastatic disease post-resection, current imaging modalities miss a large proportion of the microdeposits of tumour that are actually present^2,10^.

Unlike imaging, immune recognition of tumours can occur when there is no detectable lesion present. In murine models, neo-antigen specific anti-tumour CD8 T cells are generated in response to very low levels of tumour antigen^11,12^ and in fact, tumour specific T cell responses can be detected even when subcutaneous tumours are completely impalpable^13^. Therefore, tumour-specific CD8 T cells could be a sensitive marker for the presence of metastatic disease. On the other hand, any such T cells detected could simply be a memory population, driven by the original tumour that was resected and not reflective of the presence of metastases at all.

It is now considered most likely that the main targets for immune recognition are tumour neo-antigens (tumour-specific mutations). These neo-antigens, and the T cell (specific) response to them, can be identified in animals and patients by using advanced sequencing, bioinformatics and T cell assays^14–17^. We reason that the most sensitive method of detection of the presence of metastatic disease may be a detectable shift in neo-antigen specific CD8 T cell frequency. This could allow for greater benefit from early adjuvant immunotherapy^18^ before tumour is even detectable by imaging.

Our AB1-HA cell line is an ideal probe to study this question. AB-HA cells express the model viral neo-antigen haemagglutinin (HA). It also carries 2 natural carcinogen-induced mouse neo-antigens in Uqcrc2 and Unc45a, discovered using whole exome sequencing (WES) and RNAseq^14^(Unc45a, unpublished data). Uqcrc2 is a protein important in oxidative phosphorylation, Unc45a is a protein involved in the regulation of the actomyosin system. *In vivo* immune responses to mutated Uq2 and Unc45a are detected in AB1-HA tumours and their draining lymph nodes. In this study we compared neo-antigen specific T cell responses with PET-CT imaging to determine if the former was indeed more sensitive to the presence of metastatic disease than imaging.

We show that increased T cell responses to neo-antigen are indeed a sensitive marker of early metastatic lung disease, and that responses to a combination of several tumour specific neo-antigen T cell responses performed even better than single neo-antigen responses as an sensitive method of detection of metastatic lung disease compared to PET-CT.

## Results

### Development of a metastatic disease model

**Figure 1:**
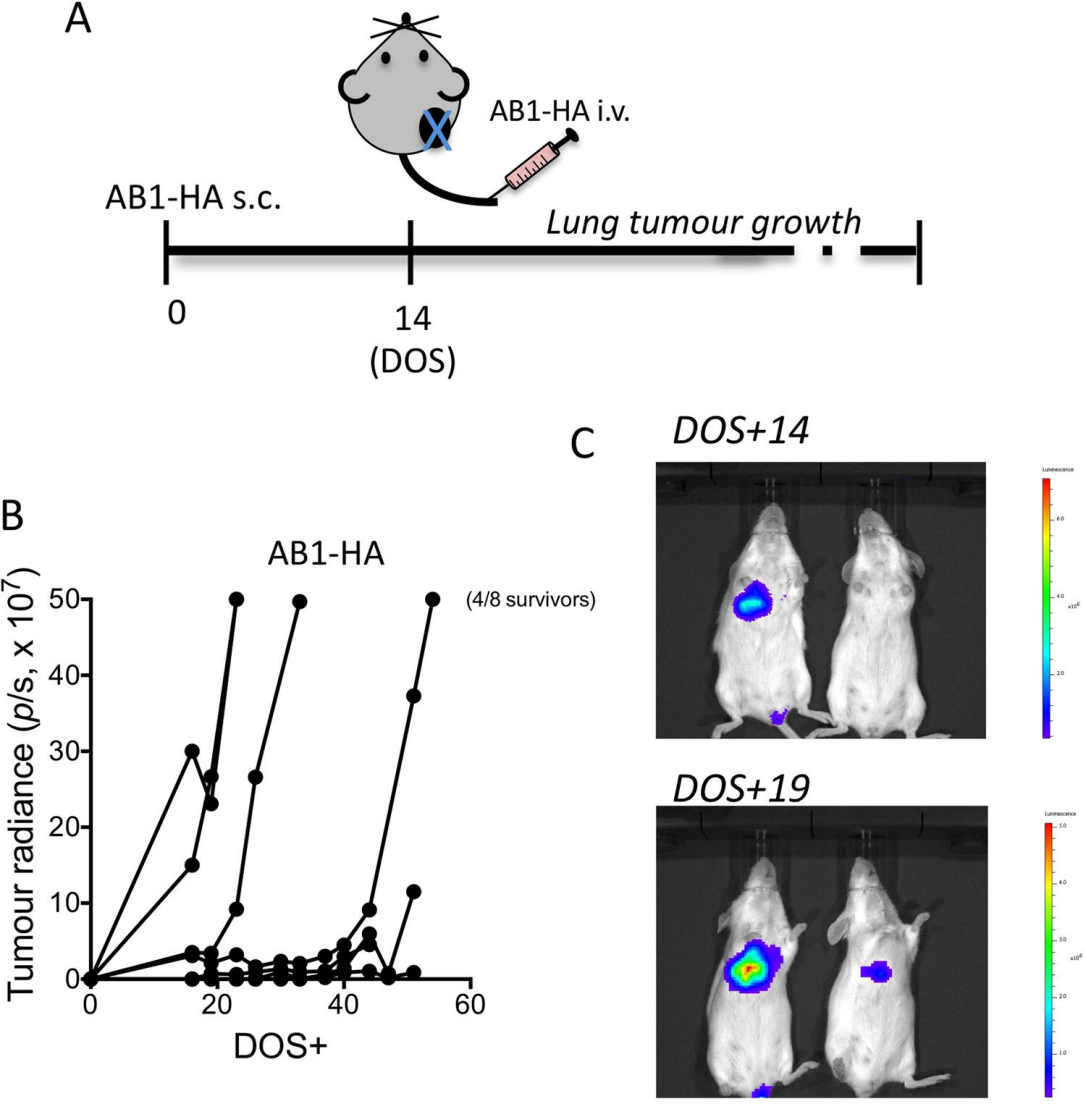
Metastatic lung disease model. Mice received 5 × 10^5^ AB1-HA cells s.c. on day 0, 1 × 10^6^ AB1-HA_LUC cell i.v. on day14, and tumours were surgically resected from all mice on day 14. Lung tumour growth was measured in the IVIS (**A**) Experimental plan. (**B**) lung tumour growth by IVIS, (**C**) Detection of tumour growth on IVIS. (D) Histology of lung tumour, H&E staining.

**Figure 2:**
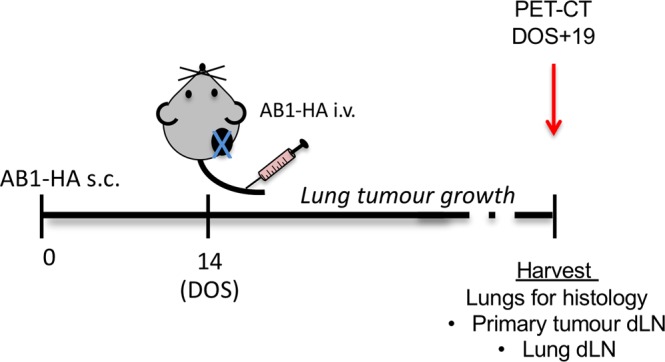
Diagrammatic depiction of metastatic disease model. Mice received 5 × 10^5^ AB1-HA cells s.c. on day 0, 1 × 10^6^ AB1-HA_LUC cell i.v. on day14, and tumours were surgically resected. Mice were ^15^FDG PET-CT imaged on day 19, and tissue harvested for analysis within 24 hours.

In order to mimic occult metastatic disease post-surgery, mice bearing subcutaneous, AB1-HA tumour underwent surgical resection of primary tumour, and on the day of surgery mice received 1 × 10^6^ AB1-HA luciferase expressing (AB1-HA_LUC) cells intravenously (i.v.; Fig. 1A). In this experimental model 62.5% of mice developed metastatic lung disease by day 50 (Fig. 1B), as determined by positive *In Vivo* Imaging Systems (IVIS) imaging (Fig. 1C). The remaining mice remained tumour free as determined by histology (data not shown). We noted that approximately half of the mice had developed metastatic lung disease by day 19 post-surgery, with tumours in the range 2.9–30.0 × 10^7^ photons/sec as determined by IVIS (Fig. 1B). Accordingly, further experiments to compare lung metastatic disease burden, by histology, to PET-CT or neo-antigen specific T cell responses were harvested at this time point, as depicted in Fig. 2.

### Neo-antigen specific T cells decline in the primary tumour draining lymph node after surgery

**Figure 3:**
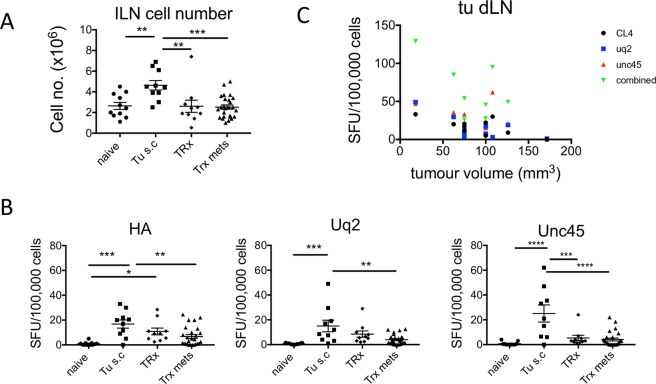
Monitoring neo-antigen specific T cells at the primary tumour site. Groups: Naïve, tumour free; TRx, 5 × 10^5^ AB1-HA cells s.c. on day 0, tumour resected day 14; Trx Mets + , 5 × 10^5^ AB1-HA cells s.c. on day 0, tumour resected on day 14, i.v. AB1-HA_LUC inoculation on DOS, with detection of metastatic disease by histology; Trx mets-, 5 × 10^5^ AB1-HA cells s.c. on day 0, tumour resected day 14, i.v. AB1-HA_LUC inoculation on DOS, with detection of metastatic disease by histology; Tu s.c, 5 × 10^5^ AB1-HA cells s.c. 14 days prior to harvest. Mice were PET-CT imaged on day 19 post-surgery, and 16 hours later dLN from the primary tumour site were analysed for neo-antigen specific T cells by ELISPOT assay. (**A**) Total cell number of lymph node cells. (**B**) Primary tumour dLN neo-antigen specific T cell frequency (SFU/100,000 cells) for peptides CL4, Uq2, and Unc45 compared to tumour volume. (**C**) Neo-antigen specific T cell frequency in the primary tumour dLN in the naïve group, healthy tumour free mice the naïve group; Tumour resection (TRx) group, surgical resection of AB1-HA subcutaneous tumour only; Tumour (Tu s.c.) group, mice bearing AB1-HA subcutaneous tumour (70 mm^3^) on the day of harvest; TRx metastatic disease (TRx mets), surgical resection of tumour with i.v. AB1-HA cells.

Next, in order to determine if neo-antigen specific T cell frequency declined after surgery, we examined the neo-antigen specific T cell response after surgical resection in the subcutaneous tumour local draining lymph nodes (Inguinal lymph node and axillary lymph node). Figure 3A indicates significantly increased cell number in the local draining lymph nodes of subcutaneous tumour bearing mice (Tu s.c.) group compared to naïve, tumour resection (TRx) and tumour resection with AB1-HA i.v. on day of surgery (TRx mets) groups. Notably, IFNγ ELISPOT indicated HA (16.80 ± 3.33 SFU/100,000 cells), Uq2 (15.05 ± 4.66 SFU/100,000 cells) and Unc45 (25.11 ± 6.94 SFU/100,000 cells) neo-antigen specific T cells were significantly increased in the Tu s.c. group compared to naïve mice (0.95 ± 0.44 SFU/100,000 cells, 0.68 ± 0.17 SFU/100,000 cells, and 0.77 ± 0.42 SFU/100,000 cells, respectively; Fig. 3B). In the TRx group Unc45 neo-antigen specific T cells (5.35 ± 2.14 SFU/100,000 cells) were significantly reduced compared to the Tu s.c. group, and neo-antigen T cell detection for HA Uq2 and Unc45 were similar to the baseline naïve group. Of note, intravenous injection of AB1-HA_LUC cells on the day of surgical resection of the subcutaneous tumour (as per Fig. 2) in the TRx mets group also had significantly reduced HA, Uq2 and Unc45 neo-antigen specific T cell response 6.77 ± 1.5 SFU/100,000 cells, 3.96 ± 0.79 SFU/100,000 cells, and 4.13 ± 1.17 SFU/100,000 cells, respectively, compared to the Tu s.c. group. These data indicate a reduction in neo-antigen specific T cell frequency in the subcutaneous tumour draining lymph node at 19 days post subcutaneous tumour resection.

Next, we examined whether subcutaneous tumour volume was proportional to neo-antigen specific T cell frequency in the local draining lymph node. We determined the correlation coefficient between tumour volume (mm^3^) and neo-antigen specific T cell frequency in the subcutaneous tumour draining lymph node using the Spearman Rho (Fig. 3C) statistical test. The Spearman Rho analysis indicated a negative correlation of −0.374, −0.085, −0.656 and −0.306 between tumour size and HA, Uq2 or Unc45 or combined neo-antigen specific T cells, p = 0.188, p = 0.774, p = 0.015, and p = 0.310, respectively, that was only significant for Unc45. This data indicated reduced frequency of Unc45 neo-antigen specific T cells with increasing tumour volume.

### Detection of lung metastatic disease by histology and PET-CT

**Figure 4:**
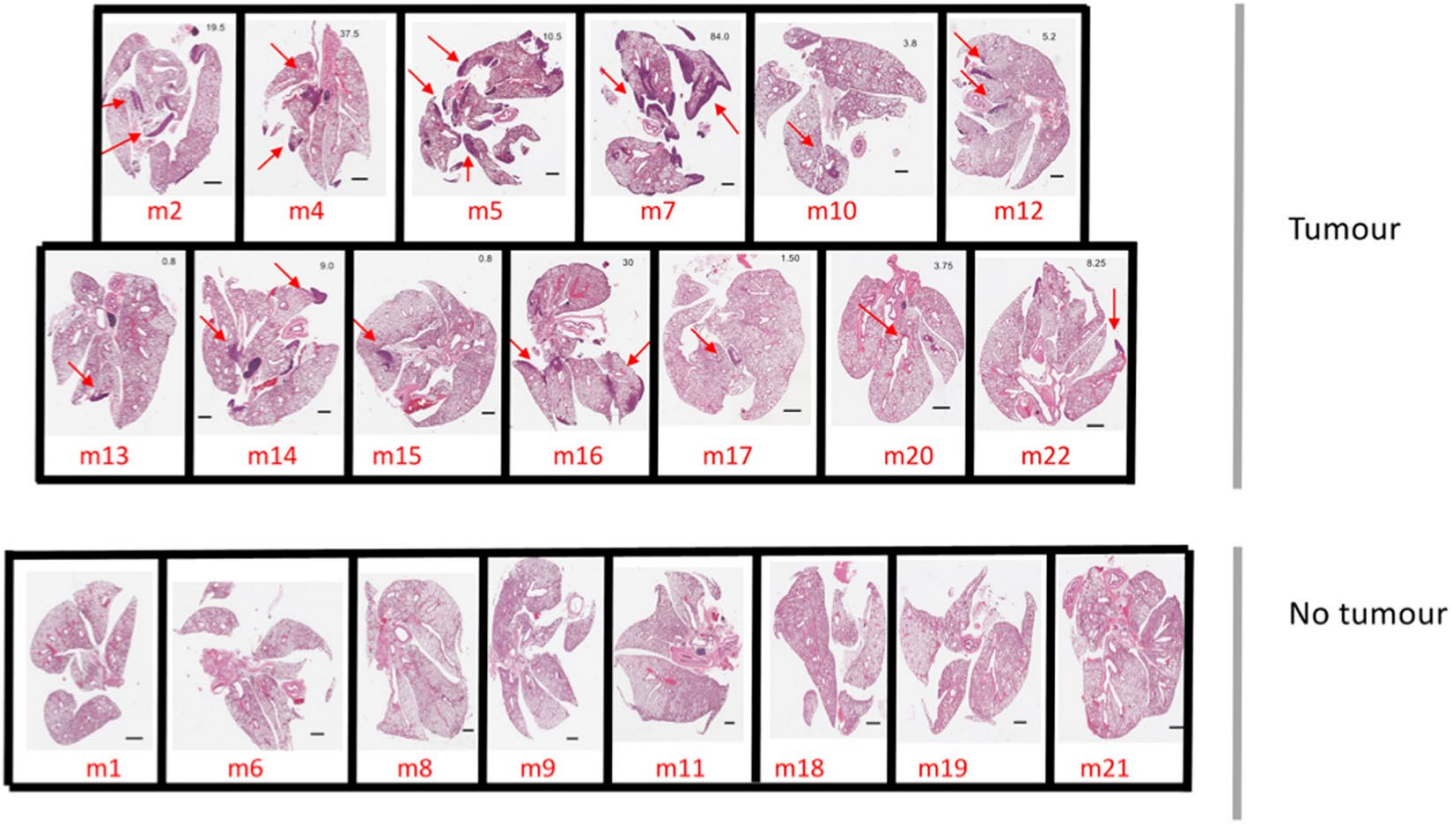
Histology for detection of metastatic disease. Mice received 5 × 10^5^ AB1-HA cells s.c. on day 0, 1 × 10^6^ AB1-HA_LUC cell i.v. on day14, and tumours were surgically resected from all mice on day 14. Mice were PET-CT imaged on day 19 after surgery. (**A**) Experiment schema. (**B**) Representative H&E slide for each mouse with tumour volume (mm^3^) indicated in top right-hand corner of image.

Next, in order to determine the limit of tumour detection by PET-CT we compared imaging with lung histology. At day 19 after surgery, mice were subject to PET-CT scanning with subsequent harvest of lung tissue for histology, and dLN and spleen for detection of tumour specific T cells (Fig. 2). In order to determine the true frequency of lung metastatic disease in all mice, serial lung section underwent histological examination (Fig. 4). Tumour was detected in 61.9% of mice (13 of 21). Next the burden of lung metastatic disease was determined for each mouse in serial lung sections and tumour volume calculated using FIJI (as described in Materials and Methods) and was in the range <0.8–84.0 mm^3^.

**Figure 5:**
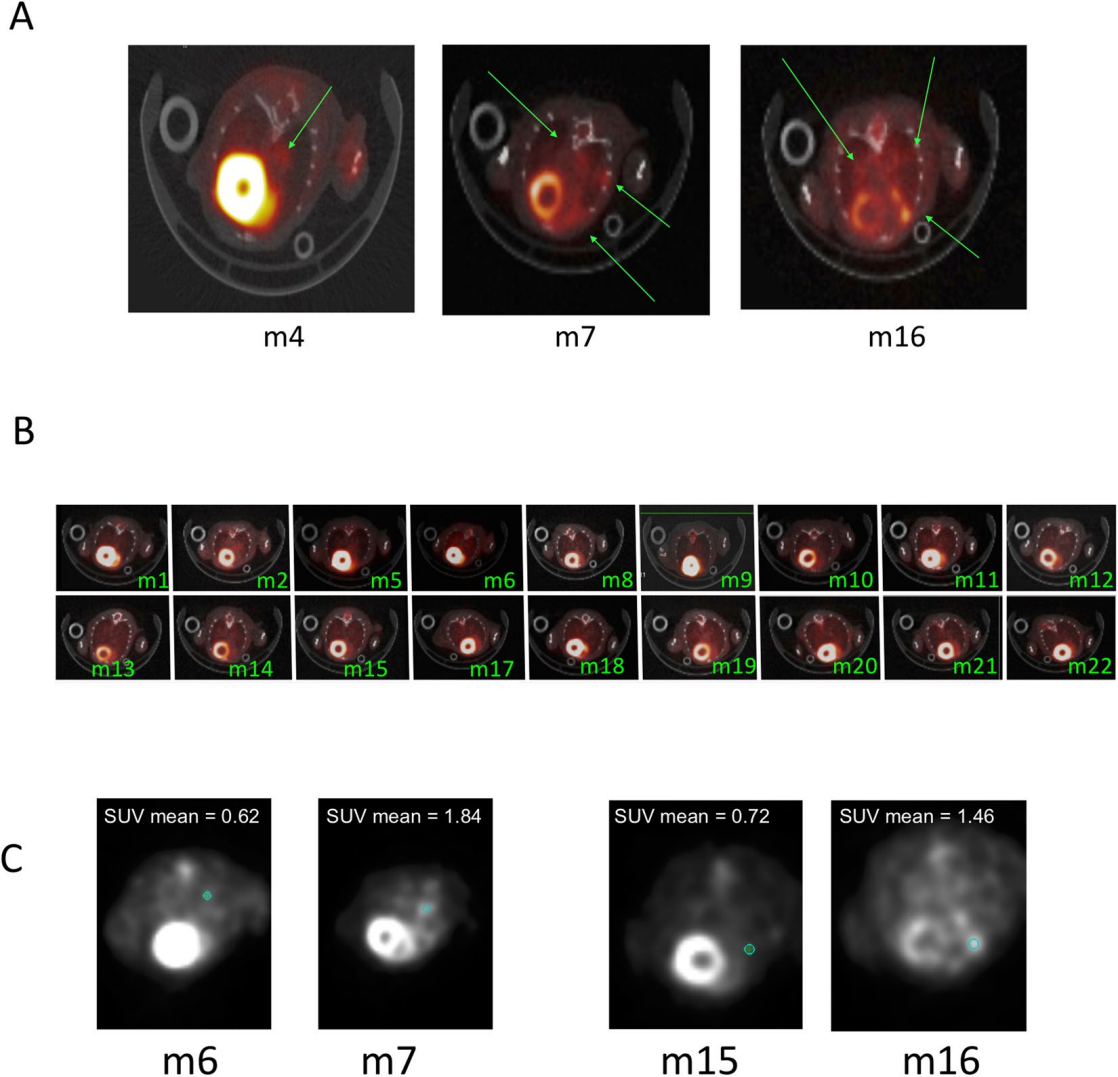
PET-CT for detection of metastatic disease and tumour burden. Mice received 5 × 10^5^ AB1-HA cells s.c. on day 0, 1 × 10^6^ AB1-HA_LUC cell i.v. on day 14, and tumours were surgically resected from all mice on day 14. Mice were PET-CT imaged on day 19 after surgery, and 16 hours later lungs were harvested, for analysis by histology. (**A**) Representative coronal sections of lung tumour showing PET-CT fused images, green arrow indicates activity. (**B**) PET-CT lung tumour negative PET-CT images. (**C**) Matched SUV mean (blue circle) for PET-CT negative and positive lung tumour detection.

Subsequently, we had the PET-CT images analysed by a radiologist for detection of lung metastatic disease. PET-CT images are shown in Fig. 4, and the radiographer was blinded to the histological results. Of the 21 lungs imaged by ^15^FDG PET-CT, three mice were determined as lung metastatic disease positive (Fig. 5A). The SUV_MEAN_ was determined for two PET-CT lung tumour positive scans for Mouse 7 as 1.843 ± 0.071 on a background of 0.622 ± 0.017; and Mouse 16 as 1.464 ± 0.083 on a background of 0.715 ± 0.048 (Fig. 5C). In the third mouse PET-CT fusion of images was corrupt and SUV could not be determined.

These data indicate that PET-CT detects lung metastases at >30 mm^3^, but clearly misses the majority of smaller metastatic disease deposits. Thus in animals and in humans, there can be a large burden of micrometastatic disease without it being detectable by current advanced imaging technologies.

### Neo-antigen specific T cells and lung metastatic disease

**Figure 6:**
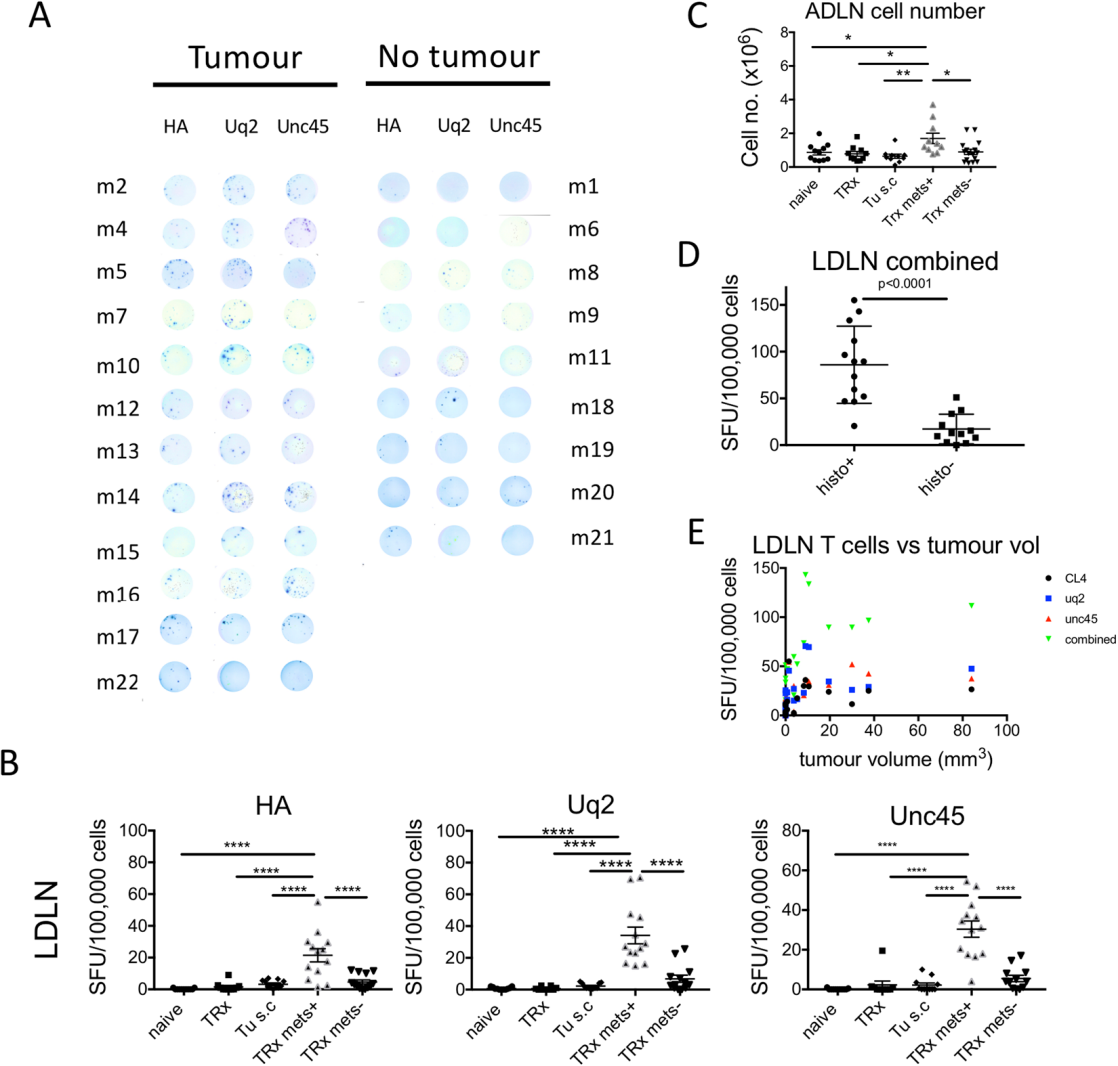
Monitoring neo-antigen specific T cells at the lung metastatic site. Groups: Naïve, tumour free mice; TRx, Mice received 5 × 10^5^ AB1-HA cells s.c. on day 0, tumour resected on day 14; Trx Mets+, Mice received 5 × 10^5^ AB1-HA cells s.c. on day 0, tumour resected on day 14, i.v. AB1-HA_LUC inoculation on DOS, with detection of metastatic disease by histology; Trx mets-, Mice received 5 × 10^5^ AB1-HA cells s.c. on day 0, tumour resected on day 14, i.v. AB1-HA_LUC inoculation on DOS, with detection of metastatic disease by histology; Tu s.c, mice received 5 × 10^5^ AB1-HA cells s.c. 14 days prior to harvest. Mice were PET-CT imaged on day 19, and 16 hours later dLN from the lung were analysed for neo-antigen specific T cells by ELISPOT assay. (**A**) IFNg ELISPOT cell assay result. (**B**) Lung dLN neo-antigen specific T cell frequency (SFU/100,000 cells) for peptides CL4, Uq2, and Unc45. (**C**) Total cell number of lymph node cells. (**D**) Combined lung dLN neo-antigen specific T cell frequency (SFU/100,000 cells) for peptides CL4, Uq2, and Unc45. Groups include the naïve group, healthy tumour free mice the naïve group; Tumour resection (TRx) group, surgical resection of AB1-HA subcutaneous tumour only; Tumour (Tu s.c.) group, mice bearing AB1-HA subcutaneous tumour (70 mm^3^) on the day of harvest; TRx metastatic disease onset (TRx mets+), surgical resection of tumour with onset of metastatic lung disease; and TRx without metastatic disease (TRx mets-), surgical resection of tumour without metastatic lung disease development. (**E**) LDLN neo-antigen specific T cell frequency compared to lung metastatic disease burden.

In order to determine if neo-antigen specific T cells were reflective of the presence of lung metastatic disease, we performed an IFNγ ELISPOT assay with lung draining lymph node cells (LDLNs; Fig. 6). Notably, mice that underwent tumour resection with metastatic disease (TRx mets + group) had significantly elevated HA (21.46 ± 4.15 SFU/100,000 cells), Uq2 (34.12 ± 5.23 SFU/100,000 cells), and Unc45 (30.38 ± 4.09 SFU/100,000 cells) neo-antigen specific T cell frequencies compared to naïve, no tumour animals (HA- 0.18 ± 0.12, Uq2−0.73 ± 0.24, and Unc45- 0.14 ± 0.10 SFU/100,000 cells, respectively, Mean ± SE; Fig. 6B). In addition, HA, Uq2 and Unc45 neo-antigen specific T cells were significantly increased in the TRx mets+ group compared to the TRx mets- group. This difference would be further elevated by the significantly increased cell number in the LDLN of lung metastatic disease mice (Fig. 6C).

### A combination of neo-antigen specific T cell responses increases sensitivity of metastasis detection

Next, we combined the frequency of HA, Uq2 and Unc45 neo-antigen specific T cells and compared metastatic lung disease and healthy lungs (Fig. 6D). Combined neo-antigen specific T cells were strikingly increased in frequency, 85.96 ± 11.45 SFU/100,000 cells for the TRx mets + group compared to 17.21 ± 4.57 SFU/100,000 cells for healthy lungs in the TRx mets- group, (p < 0.0001, unpaired two-tailed *t*-test). These data indicate that frequency of neo-antigen specific T cells increased with lung metastatic disease onset.

### Tumour burden and neo-antigen specific T cell responses

We next determined if total lung tumour burden from metastatic disease correlated with neo-antigen specific T cell frequency. Increased neo-antigen specific T cells in the LDLN positively correlated with lung metastatic disease. The Spearman Rho correlation coefficient was 0.642, 0.805, 0.813 and 0.809 comparing tumour size and HA, Uq2 or Unc45 or combined neo-antigen specific T cells, all with significance p = 0.000. (Fig. 6E).

Further, next examined the neo-antigen specific T cell response in the spleen. Neo-antigen specific T cell responses by IFΝγ ELISPOT assay were not significantly increased for either individual or combined HA, Uq2 and Unc45 compared to the naïve group (Supp. Fig. 1A). There were significantly reduced total spleen cell numbers in the TRx mets+ group compared to the naïve group, with no other significant changes between groups (Supp. Fig. 1B). No correlation between lung tumour burden and spleen neo-antigen specific T cells was observed.

These data indicate that increased neo-antigen specific T cells in the lung draining lymph nodes are an early indicator of lung metastatic disease.

## Discussion

Treatment for the early stages of NSCLC consists of surgical resection followed by adjuvant chemotherapy, immunotherapy, and/or radiotherapy. Unfortunately, the relapse rate from recurrence or onset of metastatic disease after primary lung tumour resection remains high. For example, the Stage IA/B or IIA/B the 5-year survival rates are 49/45% and 30/31%, respectively^19^. Adjuvant chemotherapy has demonstrated survival benefit in some disease stages, including high-risk Stage IB, Stage II and IIIA disease^20^, but many patients still relapse. Immunotherapy is a breakthrough modality for the treatment of a number of cancers, including NSCLC. Immune checkpoint blockade therapy has increased the 5-year survival rates in patients whose tumour has metastasised (Stage IV) to 20%^6^.

Crucially, immunotherapy is more effective when tumours are small^5^, so it is thought that early detection of metastatic disease, before tumour deposits become large, will increase treatment efficacy. This study showed that monitoring of neo-antigen specific T cell responses may be an early indicator of disease, that is more sensitive to the presence of metastatic tumour relative to current imaging modalities.

In this study we tested the sensitivity of PET-CT, an imaging technique that combines functional and anatomical information in a non-invasive fashion^21^, in our animal model. Although PET-CT imaging detected metastatic lung disease in the range 3–4 mm (>30 mm^3^), it missed metastatic disease deposits. This is consistent with the clinical setting in which, the limits of PET-CT detection of tumour in patients are in the order 6–7 mm (>216 mm^3^). Although a useful diagnostic tool, the potential to miss small metastatic lesions, along with the known lack of sensitivity^22^ and potential for significant radiation exposure associated with sequential scans, highlight the practical limitations of PET-CT and the need for additional disease biomarkers to be investigated.

Due to advances in next generation sequencing, bioinformatics and peptide binding algorithms, it has recently become possible to track tumour neo-antigen specific T cell responses to cancer, i.e. responses to the mutations that are present within each tumour^14,23–26^. One advantage of the tumour neo-antigen specific T cell response is that they are truly unique to the tumour tissue, and therefore will not have undergone prior clonal deletion or tolerance in the thymus^16^, in contrast to tumour associated antigens (TAAs), and germline cancer antigens (CGAs). The selective expression of neo-antigens on tumour cells strategically places neo-antigen specific T cells as a biological marker to reflect tumour burden. Limitations in the clinical application of personalised neo-antigen based therapies include limitations in the capacity of current genomics approaches to identify all bona-fide neo-antigens using current *in silico* prediction algorithms. Accordingly, development of new methods for the rapid and more accurate identification of neo-antigens on tumour cells are a current research focus in cancer^27^. It is also possible that immune responses against other tumour antigens (self, differentiation, common mutations) could offer an early diagnostic marker of metastasis, but this needs to be further tested.

Neo-antigen specific T cells are not just memory cells but reflect the presence of tumour: We show that surgical resection of the primary subcutaneous tumour reduced the frequency of neo-antigen specific T cells specific for all three neo-antigens HA, Uq2 and Unc45 when examined in the primary tumour dLN 19 days post-surgery. We have previously shown, using T cell proliferation assays in the Lyons-parish assay, that HA-specific T cells in the draining lymph node steadily decline after surgical resection, reaching non-detectable levels by day 14 post-surgery^28^. In humans, melanoma neo-antigen specific T cells delivered as adoptive cell therapy contract in number following complete tumour regression^29^.

In this study HA, Uq2 and Unc45 neo-antigen specific T cell response declined with tumour resection alone, however, when occult tumour cells were introduced on the day of surgery, residual neo-antigen specific T cells in the tumour draining lymph nodes remained. Taken together, the presence of tumour antigen might be driving the continuous generation of neo-antigen specific T cells in the tumour draining lymph nodes, and as such removal of the primary tumour leads to a reduction in neo-antigen specific T cells.

In this paper we further demonstrate lung draining lymph node neo-antigen specific T cell response post-surgery correlates with onset of metastatic disease. Tumour specific CD8 T cells are activated (‘cross-primed’) in dLNs in a process that is sensitive to low amounts of total tumour antigen, around 200 pg^11^. We demonstrate here that neo-antigen specific T cell responses in the LDLN of mice can be detected with metastasis as small as 0.8 mm^3^. This is substantially more sensitive than PET-CT imaging in terms of the limits of detection.

Further, combinations of neo-antigen reactivities are more sensitive to the presence of tumour than responses to single neo-antigens. In this study we observed variations in HA, Uq2 and Unc45 immunoreactivity between individual mice, and in many cases a particular neo-antigen dominated the response to metastatic disease. This is a common finding in individual animals, even when the strain and tumour are identical [Ma S *et al*., unpublished data]. Other studies have also shown that neo-antigen specific T cell responses correlate more closely with tumour volume according to neo-antigen type or antigen expression levels on the tumour surface^7,11^. Our data suggest that monitoring of more than one neo-antigen specific T cell response may be necessary for optimal, reliable detection of metastatic disease. This notion takes on extra importance in the knowledge that neo-antigen response are dynamic in tumour evolution. For example, tracking of neo-antigen T cell responses in melanoma patients indicates T cell driven immune-editing of tumour^29^ and in an ovarian cancer patient, detection of T cell response to a neo-antigen HSDL1^L25V^was seen at first recurrence but not in primary or secondary recurrence tumour tissue^15^. This indicates that changes in the neo-antigen T cell response and expressed tumour neo-antigens can occur during metastasis and/or recurrence. Studies also indicate tumour cell heterogeneity may be found when comparing the primary tumour to metastatic disease sites^15^. This means that the same neo-antigen may be expressed on some, but not all, cancer cells. Further tumour cells may suppress their expression of immunologically targeted neo-antigens, or neo-antigen specific T cells may become tolerogenic or exhausted^30^, though recent studies in melanoma have shown that neo-antigen specific T cell populations remained similar in the primary tumour compared to metastatic disease sites^31^. Furthermore, two neo-antigen specific T cell populations were responsible for killing 90% of autologous melanoma cells^31^. Here, we tracked 3 tumour specific neo-antigens T cell response that were individually predictive of metastatic disease. Importantly, in this study the combination HA, Uq2 and Unc45 neo-antigen specific T cell responses was most strongly predictive of lung metastatic disease. This suggests that patients will require careful examination of the neoantigen load in the primary resected tumour and a number of such neoantigens used to monitor the patient for recurrence.

In this study model, we determine disease recurrence via neo-antigen specific T cells generated to primary tumours by the immune system. A better model may be orthotopically inoculated primary tumour however lung tumour resection in mice is not always feasible. In addition, orthotopic models and/or spontaneous metastases models often have poor reproducibility. Importantly, we have been able to establish a murine metastatic disease model that stimulates neo-antigen specific immune response to primary tumour, in order to determine relevance to metastatic disease.

Finally, given that we focussed our attention on the dLN, which cannot be easily examined when following up patients, we also examined neoantigen responses in the spleen, as large volume blood samples are impossible to obtain in mice. We did not find the same close correlation with metastasis that we found in the dLNs. This does not mean that sequential bloods, which are the easiest to obtain, will not provide the relevant early information in metastatic disease, because the spleen is a secondary lymphoid organ, not the blood. Only sequential studies in patients’ post-surgery will accurately test the implications of our animal study. We currently have patient studies in progress wherein we collect lymph node and blood samples, for subsequent analysis of neoantigen responses to correlate with PET-CT imaging, after lung cancer surgery (RGS0000001516). Although blood sampling will be studied, sequential lymph node sampling via endobronchial ultrasound (EBUS) sampling for neo-antigen specific T cell fluctuations may also be a powerful tool for early diagnosis of metastasis.

In summary, we show increased neo-antigen specific T cells in response to very small deposits of tumour growth in the lung. Indeed, low burden lung metastatic disease showed a strong correlation with HA, Uq2 and Unc45 neo-antigen specific T cell frequency in the LDLNs. The specific neo-antigen specific T cells responses to the same tumour neo-antigen can vary between individuals but combined multi-neo-antigen specific T cell responses were most strongly predictive of metastatic disease. These observations have major translational implications. If verified in humans, sequential tracking of neoantigen responses may enable clinicians to begin adjuvant therapy much earlier than otherwise possible, with the prospect of improving the survival rate in patients.

## Methods

### Mice

Eight-week old female BALB/c mice were obtained from the Animal Resource Centre (Murdoch, WA, Australia) and housed at the Harry Perkins Bioresources Facility (Nedlands, WA, Australia). All mice were maintained under standard specific pathogen free housing conditions and animal experiments were conducted in accordance with the Australian code for the care and use of animals for scientific purposes, 8^th^ edition, 2013 and the University of Western Australia animal ethics and guidelines protocols (Harry Perkins Institute of Medical Research Animal Ethics Committee approved, protocol AE049).

### Tumour cell culture and inoculation

The murine mesothelioma cell line AB1-HA cell line was generated by inoculating crocidolite asbestos intraperitoneally (i.p.) in BALB/c mice, collecting the peritoneal exudate and passaging cells *in vivo* and *in vitro* until a stable, clonal cell line was obtained^32^. The AB1 cell line was then transfected with influenza hemagglutinin (HA) from the Mt Sinai strain of PR8/34/H1N1 influenza virus to generate the well characterised AB1-HA cell line^13^, which was further transfected to express the luciferase expressing cell line AB-HA_LUC.

### Tumour metastatic disease model

Mice received 5 × 10^5^ AB1-HA cells on day 0, tumour growth was monitored with callipers, and tumour surgically resected on day 14. On the day of surgery mice received intravenous injection of AB1-HA_LUC cells. Lung tumour growth was determined by IVIS, PET-CT, and/or histology at the indicated time points.

### Surgical resection of tumours

Animals were administered analgesic buprenorphine s.c. (0.1 mg/kg) at 30 min prior to surgery. Anaesthesia in mice was inducted with 4% isofluorane in 100% oxygen and maintained on at 2% isofluorane, at a flow rate of 2 L/min for the duration of surgery. Resections were performed by elliptical incisions over subcutaneous tumours spanning twice the length of the lesion, with at least 3 mm lateral margins. Tumours were dissected clear of the adjacent fascia, and wounds were closed with 7 mm wound clips (ABLE Scientific, Western Australia) or uninterrupted sutures (dissolvable 5–0 vicryl sutures, polyglactin 910, Ethicon Australia). After surgery mice received buprenorphine at 12 and 24 h^33^.

### IVIS

Mice were injected i.p. with 100 µl Luciferin (15 mg/ml; Sigma Aldrich), according to the manufacturer’s instructions. Mice were imaged for peak bioluminescence to 25 min in a Lumina II Imager (Caliper Life Sciences, Hopkinton, MA, USA).

### PET-CT

Mice were fasted for at least 6 hours, prior to anaesthesia and intravenous injection 15-FDG (7–15 MBq) and remained under 2% isofluorane for an uptake period of 60 min prior to scanning on the Bioscan BioPet/CT 105 camera, with respiratory monitoring.

All animal imaging was performed at the Australian Cancer Research Foundation, Cancer Imaging Facility at the Harry Perkins Institute of Medical Research, Perth, Australia. A Bioscan, hybrid PET/CT preclinical scanner was used to obtain images after intraperitoneal injection of mice with 18-Fluorine fluorodeoxyglucose (FDG). A one hour uptake period was performed under 2% isoflurane in 100% oxygen at a flow rate of 2 L/min.

PET/CT images were taken, following the 1 hour uptake phase, consisting of 1 emission bed position with the chest in the centre of the FOV. A 15 minute emission scan was acquired with a 250–700 KeV energy window followed by a CT, 40 kV tube voltage and 139.5 uA tube current, covering the PET FOV. PET images were reconstructed using 2D and 3D OSEM algorithms. 3D OSEM using 1 iteration and 80 subsets and 2D OSEM using 2 iterations and 16 subsets both with random and scatter correction. SUV semi-quantitative analysis was performed.

### Histology

Lungs from experimental mice were taken at day 20 and fixed in 10% formalin, and processed as paraffin embedded blocks. Lungs were serially sectioned every 750 microns onto glass slides and stained with Hematoxylin and Eosin (H&E). Slides were viewed using Aperio Scanscope XT 20x imaging (Olympus UPlanSApo 20x NA0.75 objective; Leica Biosystems, Victoria, Australia), and ImageScope software was used for image analysis. Tumour volume was determined in serial sections for each mouse using FIJI software and a 1000 micron grid.

### Preparation of single cell suspensions

Single cell suspensions were prepared from draining lymph node (dLN) upon harvest as described^34^. In brief, inguinal lymph node (ILN), axillary lymph node (AxLN), lung draining lymph nodes (LDLN; parathymic and/or upper paratracheal) were subject to digestion with type IV collagenase (1/5 mg/mL); Worthington Biochemical, Lakewood, NJ) with type I DNase (0.1 mg/mL; Sigma). Once digested, samples were centrifuged, washed, filtered through 100 µm filters and resuspended in PBS with 2% FCS.

### Neo-antigen specific T cell detection

ELISPOT assays were performed as previous^14^ on fresh single cells. In brief, 1 × 10^5^ cells were incubated in a microtitre plate pre-coated with 1 µg/ml IFNγ (clone AN18, Mab tech, Preston, Australia), with 1 µg/ml peptide (Uq2: SYMAPSTVL, Unc45a: IYEVVRSLV or HA: IYVSTVASSL; Mimetopes, Notting Hill, Australia), 1 µg/mL CD3 (Raybiotech, GA), or media alone. Cells were incubated 20 h at 37 °C and cytokine secretion detected with 1 µg/ml biotinylated IFNγ antibody (clone R4-6A2; Mabtech). Plates were counted on the Autoimmune Diagnostic AID ELISPOT reader system with count algorithm.3.2.x. The mean background SFU/10^5^ cells was subtracted from the test wells.

### Statistical analysis

Results are mean and standard error of the mean (±SEM) unless otherwise stated. ONE-way ANOVA, with Sidaks correction for multiple testing was used to determine significance between groups. Unpaired, two-tailed, T-test was used to measure significance between two groups. Linear regression coefficient and significance were determined using Spearman rho. All analysis was performed using GraphPad Prism version 7 software (GraphPad Software Inc., La Jolla, CA) or SPSS* statistics version 22 (Armonk, NY).

## Supplementary information

Supplementary Figure 1
