# Supplementary material for: Neo-antigen specific T cell responses indicate the presence of metastases before imaging
Source: Sci Rep. 2019 Oct 10;9:14640. doi: 10.1038/s41598-019-51317-3 (PMC6787183; doi:10.1038/s41598-019-51317-3)
Supplement: Supplementary file 1 — Supplementary Figure 1 [file 41598_2019_51317_MOESM1_ESM.pdf]

Neo-antigen specific T cell responses indicate the presence of metastases before imaging.

Fear VS, Forbes CA, Chee J, Ma S, Neeve S, Celliers L, Fisher SA, Dick I, Creaney J, Robinson BWS.

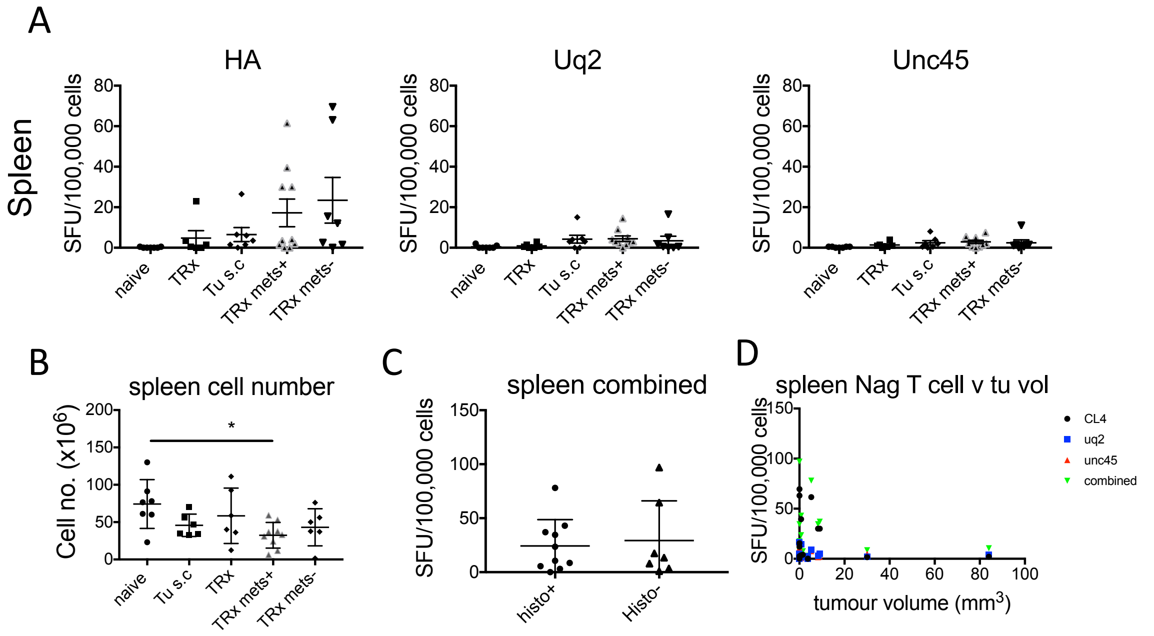

Supp Figure 1. Monitoring neo-antigen specific T cells at the lung metastatic site.

Groups: Naïve, tumour free mice; TRx, Mice received 5 x 10^5 AB1-HA cells s.c. on day 0, tumour resected on day 14; Trx Mets+, Mice received 5 x 10^5 AB1-HA cells s.c. on day 0, tumour resected on day 14, i.v. AB1-HA_LUC inoculation on DOS, with detection of metastatic disease by histology; Trx mets-, Mice received 5 x 10^5 AB1-HA cells s.c. on day 0, tumour resected on day 14, i.v. AB1-HA_LUC inoculation on DOS, with detection of metastatic disease by histology; Tu s.c, mice received 5 x 10^5 AB1-HA cells s.c. 14 days prior to harvest. Mice were PET-CT imaged on day 19, and 16 hours later dLN from the lung were analysed for neo-antigen specific T cells by ELISPOT assay. (A) Total cell number of lymph node cells. (B) primary tumour dLN neo-antigen specific T cell frequency (SFU/100,000 cells) for peptides CL4, Uq2, and Unc45. (C) Lung dLN neo-antigen specific T cell frequency (SFU/100,000 cells) for peptides CL4, Uq2, and Unc45. Groups include the naïve group, healthy tumour free mice the naïve group; Tumour resection (TRx) group, surgical resection of AB1-HA subcutaneous tumour only; Tumour (Tu s.c.) group, mice bearing AB1-HA subcutaneous tumour ($70mm^3$) on the day of harvest; TRx metastatic disease onset (TRx mets+), surgical resection of tumour with onset of metastatic lung disease; and TRx without metastatic disease (TRx mets-), surgical resection of tumour without metastatic lung disease development. (D) Spleen, neo-antigen specific T cell frequency compared to tumour volume.
